# Defective formyl peptide receptor 2/3 and annexin A1 expressions associated with M2a polarization of blood immune cells in patients with chronic obstructive pulmonary disease

**DOI:** 10.1186/s12967-018-1435-5

**Published:** 2018-03-15

**Authors:** Yung-Che Chen, Meng-Chih Lin, Chih-Hung Lee, Shih-Feng Liu, Chin-Chou Wang, Wen-Feng Fang, Tung-Ying Chao, Chao-Chien Wu, Yu-Feng Wei, Huang-Chih Chang, Chia-Cheng Tsen, Hung-Chen Chen, Taiwan Clinical Trial Consortium of Respiratory Disease (TCORE) group

**Affiliations:** 1 Division of Pulmonary and Critical Care Medicine, Kaohsiung Chang Gung Memorial Hospital and Chang Gung University College of Medicine, Kaohsiung, Taiwan; 2 Medical Department, College of Medicine, Chang Gung University, Taoyuan, Taiwan; 3 Department of Dermatology, Kaohsiung Chang Gung Memorial Hospital and Chang Gung University College of Medicine, Kaohsiung, Taiwan; 4 Department of Respiratory Therapy, Kaohsiung Chang Gung Memorial Hospital and Chang Gung University College of Medicine, Kaohsiung, Taiwan; 5 Chang Gung University of Science and Technology, Chia-yi, Taiwan; 6 Department of Internal Medicine, E-Da Hospital, I-Shou University, Kaohsiung, Taiwan; 7 Division of Pulmonary and Critical Care Medicine, Department of Medicine, Kaohsiung Chang Gung Memorial Hospital and Chang Gung University College of Medicine, 123, Ta-Pei Rd, Niao-Sung District, Kaohsiung, Taiwan

**Keywords:** Formyl peptide receptor 1/2/3, M2a polarization, Chronic obstructive pulmonary disease, Cigarette smoking, Annexin A1

## Abstract

**Background:**

Controversy exists in previous studies on macrophage M1/M2 polarization in chronic obstructive pulmonary disease (COPD). We hypothesized that formyl peptide receptor (FPR), a marker of efferocytosis and mediator of M1/M2 polarization, may be involved in the development of COPD.

**Methods:**

We examined FPR 1/2/3 expressions of blood M1/M2a monocyte, neutrophil, natural killer (NK) cell, NK T cell, T helper (Th) cell, and T cytotoxic (Tc) cell by flowcytometry method in 40 patients with cigarette smoking-related COPD and 16 healthy non-smokers. Serum levels of five FPR ligands were measured by ELISA method.

**Results:**

The COPD patients had lower M2a percentage and higher percentages of NK, NK T, Th, and Tc cells than the healthy non-smokers. FPR2 expressions on Th/Tc cells, FPR3 expressions of M1, M2a, NK, NK T, Th, and Tc cells, and serum annexin A1 (an endogenous FPR2 ligand) levels were all decreased in the COPD patients as compared with that in the healthy non-smokers. FPR1 expression on neutrophil was increased in the COPD patient with a high MMRC dyspnea scale, while FPR2 expression on neutrophil and annexin A1 were both decreased in the COPD patients with a history of frequent moderate exacerbation (≥ 2 events in the past 1 year). In 10 COPD patients whose blood samples were collected again after 1-year treatment, M2a percentage, FPR3 expressions of M1/NK/Th cells, FPR2 expression on Th cell, and FPR1 expression on neutrophil were all reversed to normal, in parallel with partial improvement in small airway dysfunction.

**Conclusions:**

Our findings provide evidence for defective FPR2/3 and annexin A1 expressions that, associated with decreased M2a polarization, might be involved in the development of cigarette smoking induced persistent airflow limitation in COPD.

## Background

Chronic obstructive pulmonary disease (COPD) is the third-ranked cause of death worldwide, killing more than 120,000 individuals each year. COPD is characterized by a T helper (Th)1 response, depression of natural killer (NK) cell activity, neutrophil- and macrophage-driven inflammation [1]. Neutrophilic inflammation persists in smoking-related COPD despite best current therapies and is particularly resistant to inhaled glucocorticosteroids, leading to progressive and irreversible airflow limitation. Inhibiting excessive inflammation is also challenging in COPD as many pathogen recognition receptors can initiate migration and the targeting of downstream signaling molecules may compromise essential host defense mechanisms [2].

M1 activation (M1 polarization), resulting in pro-inflammatory macrophages characterized by high production of pro-inflammatory cytokines, oxidative stress, strong microbicidal and tumoricidal activity, while alternative M2 activation (M2 polarization), resulting in macrophages that are considered to have anti-inflammatory, wound-healing properties, with high expression of scavenger receptors, and good efferocytosis [3]. Controversy exists in previous studies on macrophage M1/M2 polarization of COPD, indicating the needs of further investigation to clarify the role of M1/M2 polarization in this disease. Formyl peptide receptors (FPRs), essential markers of efferocytosis, belong to the seven-transmembrane G protein-coupled receptor superfamily and also a family mediate leukocyte response during inflammation, encompassing three subtypes: FPR1, FPR2 (ALX, Aspirin-triggered Lipoxin, or LXA4 receptor), and FPR3 [4]. It has been shown that FPR2/FPR2 homodimerization triggers the p38/MAPKAPK/Hsp27 pathway associated with anti-inflammatory responses, such as interleukin (IL)-10 generation, whereas PFR1/FPR2 heterodimerization triggers JNK pathway associated the delay of neutrophil apoptosis and the induction of pro-inflammatory responses [5]. Previous studies have demonstrated that FPR1 maintains M1 polarization, and that FPR2 homo-dimerization can limit M2 polarization [6]. FPR1 has been demonstrated to be an important receptor in COPD, because genetic ablation of this receptor confers protection from the development of cigarette smoke-induced emphysema in the mouse model [7]. Several known FPR2 endogenous agonists promoting inflammatory biological functions are enriched in COPD, and include serum amyloid A (SAA) and cathelicidin (LL-37) [8]. SAA has been shown to be a mediator of glucocorticoid-resistant lung inflammation that can overwhelm organ protective signaling by LXA4 at FPR2 receptors in a murine model of COPD [9]. Increased levels of LL-37 in airway epithelium after cigarette smoke exposure could stimulate collagen production in the underlying lung fibroblasts and may contribute to small airway remodeling in COPD [10]. In contrast, decreased levels of LXA4 and annexin A1 (ANXA1) have been found in pediatric patients with asthma and wheezy infants [11, 12]. Resolvin D1 (RvD1) has been reported to attenuate smoking-induced emphysema in a mouse model by reducing inflammation and promoting tissue regeneration [13]. All these three ligands have been shown to contribute to resolution of inflammation through FPR2 [14]. FPR1 and FPR2 were evenly distributed at the plasma membrane, while FPR3 was localized in small intracellular vesicles resulting from a constitutive internalization [15]. Little is known about the role of FPR1/2/3 in the development of COPD and its clinical phenotypes.

We hypothesized that there are altered FPR1/FPR2/FPR3 expressions of blood immune cells, imbalanced M1/M2 monocyte, and differential expressions of five FPR ligands, including SAA, LL37, LXA4, ANXA1, and RvD1, in the systemic inflammatory environment of COPD, opposing protective anti-inflammatory and pro-resolution pathways [4]. These insights would open the possibility of targeting FPR1/FPR2/FPR3 receptors using synthetic agonists to resolve persistent inflammation without compromising essential host defense mechanisms.

## Methods

### Subjects and clinical phenotypes in COPD patients

#### Subjects

The patient group consisted of 40 male patients with smoking related COPD, recruited from the Pulmonary Outpatient Department of Kaohsiung Chang Gung Memorial Hospital, a medical center in southern Taiwan, from October 2014 to August 2016. COPD was diagnosed on the basis of past history, physical examination, and spirometry data. The criteria of inclusion were as follows: (1) male older than 40 years of age, (2) a smoking history of ≥ 20 pack-years, (3) post-bronchodilator (BD) forced expiratory volume in one-second (FEV1)/forced expiratory vital capacity (FVC) < 70%. Those with a history of tuberculosis, asthma, bronchiectasis, or lung cancer were excluded. The control group consisted of 16 male healthy non-smokers with normal lung function and without marked chest complaint, who were recruited from the Center of Health Examination. The inclusion criteria were as follows: (1) male older than 40 years of age, (2) no past history of smoking exposure, (3) pre-BD FEV1/FVC ratio > 70%, pre-BD FEV1% predicted > 80%, and pre-BD FVC %predicted > 80%, (4) no other pulmonary disease by history and chest radiography.

### Pulmonary function test, clinical evaluation, and treatment

Pulmonary function tests were performed by experienced lung function technicians with a standard spirometer (Serial Spirotrac, Vitalograph, Buckingham, UK) according to American Thoracic Society recommendations. The best FVC measurement was recorded along with FEV1 and the FEV1/FVC calculations before and after bronchodilator (BD; fenoterol hydrobromide, Berotec^®^, Boehringer Ingelheim, 400 μg). All measurements were presented as percent predicted value. A moderate exacerbation of COPD was defined by an acute event characterized by a worsening of the patient’s respiratory symptoms that was beyond normal day-to-day variations, leading to the use of antibiotics or oral steroid. Dyspnea severity was evaluated by modified medical research council (MMRC) dyspnea scale [16]. COPD management was in accordance with the global initiative for chronic obstructive lung disease (GOLD) guidelines, which advise the use of long acting β2 agonist (LABA), long acting muscarinic antagonist (LAMA), inhaled corticosteroid (ICS), and oral theophylline [17].

### Measurement of cell surface protein expressions of FPR2/FPR1, and intracellular FPR3 of peripheral blood CD14^+^CD209^−^ M1 monocyte, CD14^+^CD209^+^ M2a monocyte, CD16^+^neutrophil, CD3^−^CD56^+^natural killer (NK) cell, CD3^+^CD56^+^ NK T cell, CD3^+^CD4^+^ helper T (Th) cell, and CD3^+^CD8^+^cytotoxic T (Tc) cell by flowcytometry

(A) Surface markers were measured by simultaneously staining for 30 min at 4 °C with directly conjugated monoclonal antibodies (mAb) as follows:
  PE Mouse Anti-Human CD56 (BD Pharmingen, USA), PE-CyTM5 Mouse Anti-Human CD3 (BD Pharmingen), CD4-PC7 (Beckman Coulter; USA), CD8-PC7, CD16-PC7, CD14-PC7 (Beckman Coulter), PerCP-Cy5.5 Mouse Anti-Human CD209 (BD Pharmingen), Anti-human CFS-conjugated FPR1 (R&D Systems; USA), PE-conjugated anti-human FPR2 (R&D Systems) or isotype control mAb.
(B) For intracellular staining, cells were stained with anti-CD8a-PE-Cyanine5 and incubated with Cytofix/Cytoperm TM for 20 min at 4 °C, and washed with Perm/Wash Buffer. Then, cells were incubated with PE anti-human FPR3 (BioLegend; San Diego, CA) or isotype control mAb.
(C) Analysis of 50,000 events was performed on Cytomics™ FC500 (Beckman Coulter; USA) using a dual staining protocol. These were further analyzed for expressions of FPR1 and FPR2 on CD16^+^ neutrophil, CD14^+^CD209^+^ M2a monocyte, CD14^+^CD209^−^ M1 monocyte, CD3^+^CD4^+^ T helper (Th) cell, CD3^+^CD8^+^ T cytotoxic (Tc) cell, CD3^−^CD56^+^ NK cell, and CD3^+^CD56^+^ NK T cell in the FL1 and FL2 channels, respectively. Intracellular FPR3 expression of these various blood immune cells was analyzed separately. Data were presented as mean fluorescence intensity (MFI), which were corrected for background fluorescence with the corresponding isotype controls.

### Measurements of SAA, LL-37, LXA4, ANXA1 and RvD1 in the serum

SAA, LL-37, LXA4, ANXA1 and RvD1 levels were assessed by a commercial enzyme-linked immunosorbent assay method (INVITROGEN, USA; HYCULTBIOTECH, USA; cusabio, China; ASSAYPRO, USA; CAYMAN, USA).

### Statistical analysis

Data were expressed as the mean ± standard deviation. One-way analysis of variance ANOVA test followed by post hoc analysis with Bonferroni test was used for comparing mean values of more than two experimental groups in case of normal and homogeneous data, while Brown–Forsythe test followed by post hoc analysis with Tamhane’s T2 test was used in case of normal and non-homogeneous data. Student t-test, Mann–Whitney U test, or paired t-test was used for comparing continuous values of two experimental groups, where appropriate. Categorical variables were analyzed using Chi square test. To investigate independent biomarkers associated with COPD and generate coefficient with 95% confidence intervals (CI), variables that showed significant differences between COPD patients and healthy non-smokers, as well as age, were all put into a stepwise forward logistic regression analysis. Multiple linear regression analysis was used to minimize the effects of confounding factors on the comparisons of continuous variables, and to provide an adjusted p value. A p-value of less than 0.05 is considered statistically significant.

## Results

### Demographics of the participants

A total of 56 subjects, including 40 male patients with COPD and 16 male healthy non-smokers, were enrolled and analyzed. Characteristics of cases and controls are listed in Table 1. The study population was all Asian in ethnicity. Pulmonary function tests of the healthy non-smokers were normal, and significantly greater than those of the COPD patients. The COPD patients were older than the healthy non-smokers, but both groups were matched in co-morbidity and had a similar body mass index (BMI), glycohemoglobin (HbAc1), triglyceride, and total cholesterol level. The patient group included active or ex-smokers with COPD [Global Initiative for Obstructive Lung Disease (GOLD) stage I–III]: 7 (17.5%) GOLD I, 21 (52.5%) GOLD II, 12 (30%) GOLD III.

**Table 1:** Demographic characteristics and pulmonary function test parameters of the 40 COPD patients and 16 healthy non-smokers

	Healthy non-smokers N = 16	COPD patients at diagnosis N = 40	p value	COPD patients followed up after 1-year treatment N = 10
Age, years	53.9 ± 12.2	63.4 ± 4.5	0.009	69.5 ± 9.5
BMI, kg/m^2^	25.38 ± 4.6	25 ± 4.2	0.775	24.6 ± 4.6
Smoking exposure, pack-years	0	64.4 ± 40.1	< 0.001	65.5 ± 50.8
Ex-smokers, n (%)		21 (52.2)		7 (70%)
Diabetes mellitus, n (%)	1 (6.3)	5 (12.8)	0.478	
Stroke, n (%)	0 (0)	2 (5.1)	0.356	
Chronic hepatitis, n (%)	1 (6.3)	5 (12.8)	0.478	
Chronic kidney disease, n (%)	0 (0)	5 (12.8)	0.133	
Congestive heart failure, n (%)	0 (0)	0 (0)	1	
Myocardial infarction, n (%)	0 (0)	0 (0)	1	
MMRC score	0.38 ± 0.5	1.49 ± 0.99	< 0.001	1.2 ± 0.79
HbA1c	5.8 ± 0.5	5.9 ± 0.6	0.663	6.0 ± 0.8
Triglyceride	105.7 ± 60.4	119.7 ± 50.2	0.389	146.3 ± 82.7
Cholesterol	187.9 ± 44.4	185.7 ± 36.6	0.853	184.4 ± 38.5
Pre-BD FEV1 %predicted, %	113.18 ± 16.1	57.72 ± 24.56	< 0.001	56.74 ± 12.96
Pre-BD FEV1/FVC, %	81.56 ± 5.4	54.44 ± 15.38	< 0.001	55.03 ± 10.27
Pre-BD FEF25-75%, %predicted	109.6 ± 27.1	26.2 ± 20.4	< 0.001	21.28 ± 9.63
Post-BD FEV1, %predicted	N.A.	61.68 ± 22.68		61.7 ± 13.28
Post-BD FEV1/FVC, %	N.A.	57.1 ± 13.1		56.74 ± 11.27
Post-BD FEF25–75%, %predicted	N.A.	28.8 ± 17.8		25.37 ± 11.14

### Decreased blood M2a percentage and increased NK/NK T/Th/Tc cell percentage in COPD patients

**Fig. 1:**
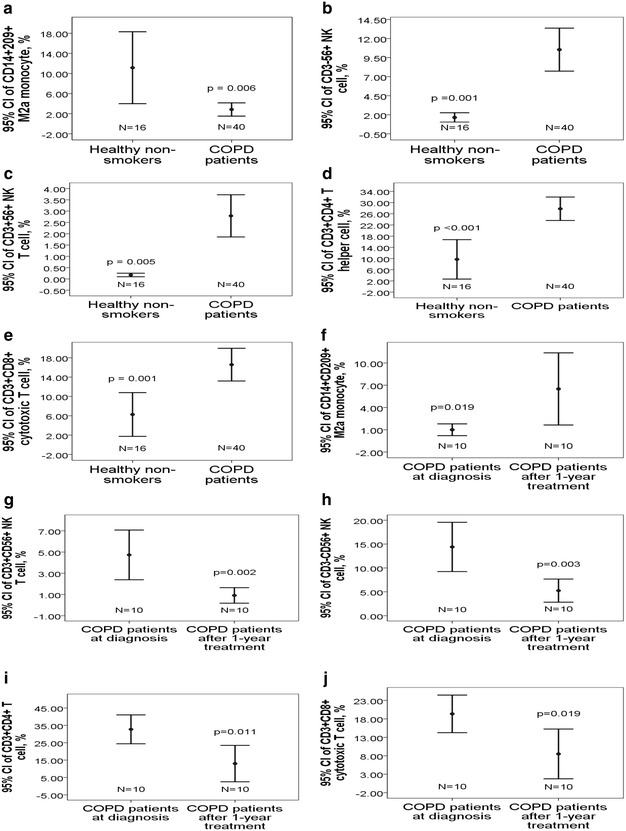
Percentages of blood immune cell subpopulations in COPD patients and healthy non-smokers. The 40 COPD patients at presentation had significantly **a** lower blood M2a percentage, **b** higher NK cell percentage, **c** higher NK T cell percentage, **d** higher T helper (Th) cell percentage, and **e** higher T cytotoxic (Tc) cell percentage as compared with the 16 healthy non-smokers. In 10 COPD patients whose blood were collected again 1 year later, **f** blood M2a percentage showed significant elevation, whereas percentage of **g** NK cell, **h** NK T cell, **i** Th cell, and **j** Tc cell showed significant reduction after treatment

**Fig. 2:**
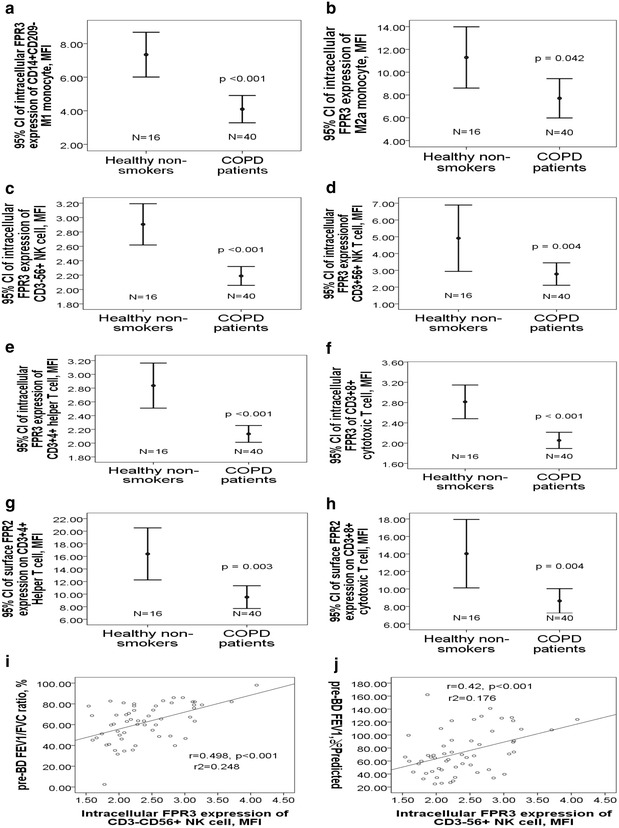
Diminished FPR2/3 expressions of blood immune cells in COPD patients. The COPD patients at presentation had significantly lower intracellular FPR3 expressions of **a** M1 monocyte, **b** M2a monocyte, **c** NK cell, **d** NK T cell, **e** Th cell, and **f** Tc cell as compared with the healthy non-smokers. Cell surface FPR2 expressions on **g** Th and **h** Tc cells were also lower in the COPD patients. FPR3 expression of NK cell was positively correlated with **i** pre-bronchodilator FEV1/FVC ration and **j** FEV1 %predicted

Blood M2a monocyte percentage in the total CD14^+^ monocytes (2.8 ± 4.1 vs. 11.2 ± 13.4%, adjusted p = 0.006, Fig. 1a) was significantly decreased, and percentages of NK (10.5 ± 8.8 vs. 1.6 ± 1.2%, adjusted p = 0.001, Fig. 1b)/NK T (2.8 ± 2.9 vs. 0.2 ± 0.1%, adjusted p = 0.005, Fig. 2c)/Th (27.8 ± 13.1 vs. 9.7 ± 13.2%, adjusted p < 0.001, Fig. 1d)/Tc (16.6 ± 10.6 vs. 6.3 ± 8.5%, adjusted p = 0.001, Fig. 1e) cells in the total lymphocytes was significantly increased in the COPD patients as compared with that in the healthy non-smokers. In 10 COPD patients whose blood samples were collected again after 1-year treatment, all these percentages were reversed to normal (all p values < 0.05, Fig. 1f–j). M2a percentage was positively correlated with intracellular FPR3 expression of M2a monocyte (r = 0.217, p = 0.03). Intracellular FPR3 expressions of NK, Th, and Tc cell were negatively correlated with percentages of NK (r = − 0.492, p < 0.001), Th (r = − 0.576, p < 0.001), and Tc (r = − 0.423, p < 0.001) cells, respectively. Cell surface FPR2 expression of Th cell was negatively correlated with Th cell percentage (r = − 0.48, p < 0.001). Amount of past cigarette smoking exposure (pack-years) were negatively correlated with M2a percentage (r = − 0.283, p = 0.005), and positively correlated with percentages of NK (r = 0.378, p < 0.001), NK T (r = 0.384, p < 0.001), Th (r = 0.403, p < 0.001), and Tc cells (r = 0.285, p = 0.005).

### Decreased FPR2/3 expressions of blood immune cells in COPD patients versus healthy non-smokers

Intracellular FPR3 expressions of M1 monocyte (4.1 ± 2.5 vs. 7.3 ± 2.5 MFI, adjusted p < 0.001, Fig. 2a) and M2a monocyte (7.7 ± 5.4 vs. 11.3 ± 5 MFI, adjusted p = 0.042, Fig. 2b) were both significantly lower in the COPD patients as compared with that in the healthy non-smokers. Intracellular FPR3 expressions of NK cell (2.2 ± 0.4 vs. 2.9 ± 0.5 MFI, adjusted p < 0.001, Fig. 2c), NK T cell (2.8 ± 2.1 vs. 4.9 ± 3.7 MFI, adjusted p = 0.004, Fig. 2d), Th cell (2.1 ± 0.4 vs. 2.8 ± 0.6 MFI, adjusted p < 0.001, Fig. 2e), and Tc cell (2.1 ± 0.5 vs. 2.8 ± 0.6 MFI, adjusted p < 0.001, Fig. 2f) were all significantly lower in the COPD patients as compared with that in the healthy non-smokers. Cell surface FPR2 expressions of both Th cell (9.5 ± 5.7 vs. 16.4 ± 7.8 MFI, adjusted p = 0.003, Fig. 2g) and Tc cell (8.6 ± 4.4 vs. 14 ± 7.3 MFI, adjusted p = 0.004, Fig. 2h) were significantly lower in the COPD patients as compared with that in the healthy non-smokers. Amount of past cigarette exposure was negatively correlated with FPR2 expression on Th cell (r = − 0.397, p < 0.001), FPR2 expression on Tc cell (r = − 0.329, p = 0.001), FPR3 expression of M2a (r = − 0.271, p = 0.008), FPR3 expression of M1 (r = − 0.382, p < 0.001), FPR3 expression of NK cell (r = − 0.386, p < 0.001), FPR3 expression of NK T cell (r = − 0.246, p = 0.016), FPR3 expression of Th cell (r = − 0.296, p = 0.003), and FPR3 expression of Tc cell (r = − 0.414, p < 0.001).

Stepwise logistic regression analysis showed that intracellular FPR3 expression of NK cell (OR 0.101, 95% CI 0.013–0.778, p = 0.028) and Th cell percentage (OR 1.096, 95% CI 1.013–1.185, p = 0.022) were independently associated with COPD at presentation after adjusting for age and co-morbidity. Intracellular FPR3 expression of NK cell was positively correlated with both pre-BD FEV1/FVC ratio (r = 0.498, p < 0.001, Fig. 2i) and pre-BD FEV1 %predicted (r = 0.42, p < 0.001, Fig. 2j).

### Changes in FPR1/2/3 expressions of blood immune cells after 1-year treatment in COPD patients

**Fig. 3:**
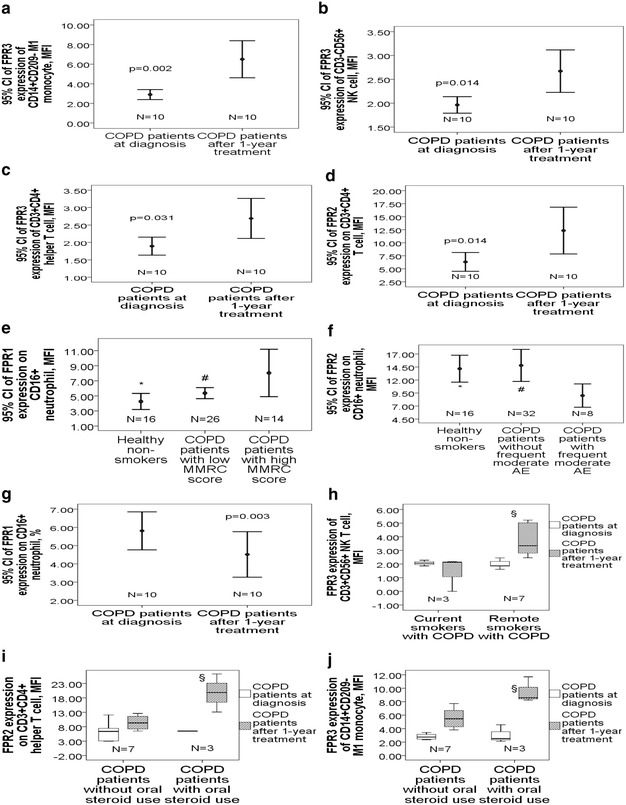
Changes in FPR1/2/3 expressions of blood immune cells in COPD patients after 1-year treatment and with various clinical phenotypes. In 10 COPD patients after 1-year treatment, **a** FPR3 expression of M1 monocyte, **b** NK cell, and **c** Th cell, as well as **d** FPR2 expression on Th cell all showed significant elevation. **e** COPD patients with a high MMRC dyspnea scale had higher FPR1 expression on neutrophil, while **f** those with a history of frequent moderate exacerbation in the past 1 year had lower FPR2 expression on neutrophil. **g** FPR1 expression on neutrophil showed significant reduction after 1-year treatment. **h** Ex-smokers with COPD had higher FPR3 expression of NK T cell than current smokers with COPD after 1 year treatment. COPD patient receiving oral steroid treatment for more than 3 months had **i** higher FPR2 expression on Th cell and **j** higher FPR3 expression of M1 monocyte than those without oral steroid use. *p < 0.05 for comparisons between COPD patients with a specific phenotype and healthy non-smokers by ANOVA test. ^#^p < 0.05 for comparison between COPD patients with and without s specific phenotype by ANOVA test. ^§^p < 0.05 for comparisons between COPD patients with and without a specific management after 1-year follow-up

In 10 COPD patients whose blood samples were collected again after 1-year treatment, intracellular FPR3 expressions of M1 monocyte (6.5 ± 2.6 vs. 2.9 ± 0.7 MFI, p = 0.002, Fig. 3a), NK cell (2.6 ± 0.6 vs. 2.0 ± 0.3 MFI, p = 0.014, Fig. 3b) and helper T cell (2.7 ± 0.8 vs. 1.9 ± 0.4 MFI, p = 0.031, Fig. 3c) all showed significant elevation. Cell surface FPR2 expression on helper T cell (12.3 ± 6.3 vs. 6.3 ± 2.5 MFI, p = 0.014, Fig. 3d) showed significant elevation after 1-year treatment. Pulmonary function tests after 1-year treatment in these 10 patients showed significant improvement in post-BD FEF25–75% %predicted (29 ± 13.8 vs. 25.4 ± 11.1%, p = 0.03) but no change in post-BD FEV1 %predicted or post-BD FEV1/FVC ratio. Among these 10 COPD patients, LAMA was used in 5 (50%), LABA in 4 (40%), ICS+LABA in 2 (20%), theophylline in 5 (50%), and oral steroid in 3 (30%).

### Comparisons of FPR1/2/3 expressions of blood immune cells among different phenotypes in COPD patients

COPD patients with a high MMRC score (≥ 2) had significantly higher cell surface FPR1 expressions on neutrophil than either those with a low MMRC score (8.0 ± 5.4 vs. 5.4 ± 1.8 MFI, p = 0.04) or healthy non-smokers (8.0 ± 5.4 vs. 4.3 ± 2 MFI, p = 0.006, Fig. 3e), while COPD patients with frequent moderate exacerbation in the past 1 year had lower FPR2 expression on neutrophil than either those without frequent moderate exacerbation (≥ 2 events in the past 1 year) (9.0 ± 2.4 vs. 14.8 ± 8.7 MFI, p = 0.007) or healthy non-smokers (9.0 ± 2.4 vs. 14.2 ± 4.9, p = 0.008, Fig. 3f). In the 10 COPD patients whose blood samples were collected again after 1-year treatment, FPR1 expression on neutrophil showed significant reduction (4.5 ± 1.7 vs. 5.8 ± 1.5 MFI, p = 0.003, Fig. 3g), while FPR2 expression on neutrophil showed elevation (18.1 ± 5.4 vs. 12.3 ± 9.8, p = 0.093) but the difference did not reach statistical significance. FPR3 expression of NK T cell showed significant elevation after 1-year treatment in the COPD patients who had quit smoking, and were higher than that in those who continued smoking (3.5 ± 1.1 vs. 1.4 ± 1.2 MFI, p = 0.031, Fig. 3h). FPR2 expression on helper T cell (19.8 ± 6.6 vs. 9.2 ± 2.3 MFI, p = 0.004, Fig. 3i) and FPR3 expression of M1 monocyte (9.5 ± 1.9 vs. 5.2 ± 1.7 MFI, p = 0.007, Fig. 3j) after 1-year treatment were both higher in COPD patients on oral steroid for more than 3 months than that in those without oral steroid use.

### Decreased serum ANXA1 levels in COPD patients and its association with a higher frequency of moderate exacerbation

**Fig. 4:**
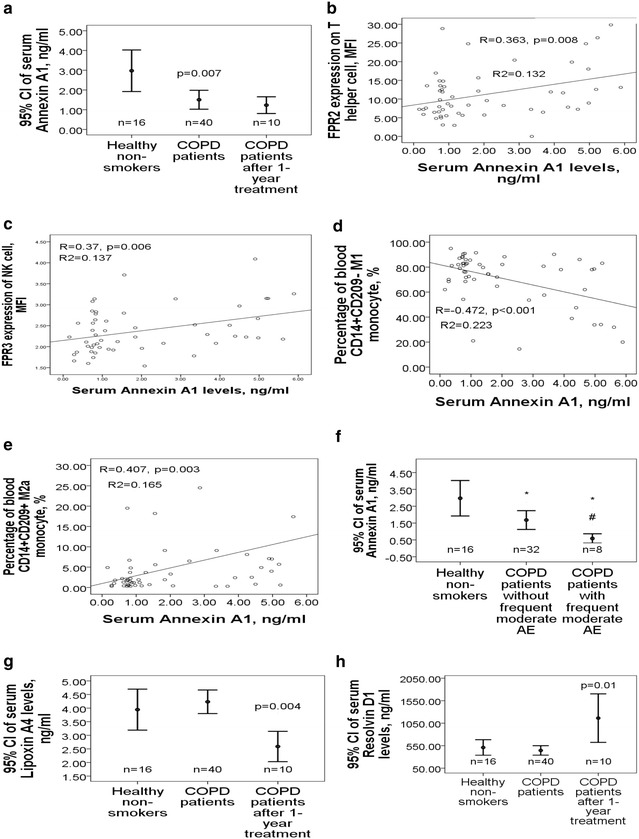
Differential serum levels of five FPR ligands, including serum amyloid A (SAA), cathelicidin (LL-37), annexin A1 (ANXA1), lipoxin A4 (LXA4), and resolving D1 (RvD1), in COPD patients. **a** The COPD patients at presentation had significantly lower serum ANXA1 levels, which remained at low levels after 1-year medical treatment. Serum ANXA1 levels were **b** positively correlated with FPR2 expression on T helper cells, **c** positively correlated with FPR3 expression of natural killer cells, **d** negatively correlated with the percentage of M1 monocyte, and **e** positively correlated with the percentage of M2a monocyte. **f** The COPD patients with a history of more than 1 moderate exacerbation in the past 1 year had significantly lower serum ANXA1 levels as compared with that in those without the history or the healthy subjects. **g** Serum LXA4 levels showed significant reduction and **h** RvD1 showed elevation after 1-year medical treatment. *p < 0.01 for comparison between COPD patients with a specific phenotype and healthy non-smokers by ANOVA test. ^#^p < 0.05 for comparison between COPD patients with and without s specific phenotype by ANOVA test

Serum ANXA1 levels was lower in the 40 COPD patients as compared with that in the 16 healthy non-smokers (1.5 ± 1.8 vs. 3 ± 1.9 ng/ml, adjusted p = 0.007, Fig. 4a), while serum SAA, LL-37, LXA4, and RvD1 levels were not different between the case and control groups. ANXA1 levels were positively correlated with post-BD FEV1/FVC (r = 0.241, p = 0.043), and pre-BD FEF25–75 %predicted (r = 0.247, p = 0.039), while negatively correlated with amount of smoke exposure (r = − 0.302, p = 0.012). Furthermore, ANXA1 levels were positively correlated with FPR2 expressions on neutrophil (r = 0.32, p = 0.019), Th cell (r = 0.363, p = 0.008, Fig. 4b), and Tc cell (r = 0.341, p = 0.012), as well as FPR3 expressions in NK cell (r = 0.37, p = 0.006, Fig. 4c), Th cell (r = 0.377, p = 0.005), and Tc cell (r = 0.428, p = 0.001). ANXA1 levels were also negatively correlated with percentages of M1 monocyte (r = − 0.472, p < 0.001, Fig. 4d), neutrophil (r = − 0.374, p = 0.006), Th cell (r = − 0.388, p = 0.004), and Tc cell (r = − 0.314, p = 0.022), while positively correlated with M2a monocyte percentage (r = 0.407, p = 0.003, Fig. 4e). Subgroup analysis showed that ANXA1 levels were further decreased in COPD patients with frequent moderate exacerbation as compared with that in those without frequent moderate exacerbation (0.58 ± 0.36 vs. 1.68 ± 1.9 ng/ml, p = 0.002, Fig. 4f) or healthy non-smokers (2.97 ± 1.9 ng/ml, p = 0.001). In the 10 COPD patients whose blood were sampled again after 1-year treatment, serum LXA4 levels showed reduction (2.6 ± 0.8 vs. 1.2 ± 0.8 ng/ml, p = 0.004, Fig. 4g), and RvD1 levels showed elevation (1163.2 ± 753.9 vs. 366.7 ± 181.8, p = 0.01, Fig. 4h), while levels of the other three FPR ligands did not change significantly.

## Discussion

For the first time, the present findings demonstrate that smoking-related COPD patients have diminished cell surface FPR2 expressions on helper and cytotoxic T cells, and diminished intracellular FPR3 expressions in M1 monocyte, M2a monocyte, NK cell, NK T cell, Th and Tc cells, associated with decreased blood M2a percentage and decreased serum levels of ANXA1. Furthermore, FPR1 expression on neutrophil was increased in COPD patients with a high MMRC dyspnea scale, whereas both FPR2 expression on neutrophil and serum ANXA1 were decreased in COPD patients with a history of frequent moderate exacerbation in the past 1 year. Moreover, several of these altered FPR1/2/3 expressions and M2a polarization, including FPR3 expression of M1 monocyte, NK cell and Th cell, FPR2 expression on Th cell, and FPR1 expression on neutrophil, were all reversed to normal after 1-year treatment and cessation of smoking.

In the present study, we found that anti-inflammatory M2a percentage was decreased in COPD patients at presentation, and reversed to normal after 1-year treatment. In line with our findings, smoking cessation has been shown to partially change the macrophage polarization towards an anti-inflammatory phenotype, CD163^+^M2c, in the periphery of the lung [18]. In addition, RvD1 inhibits cigarette smoke—induced inflammation in mice through increasing M2 macrophages and neutrophil efferocytosis [19]. In contrast, M1/M2 related gene expression analysis showed that smoking induced reprogramming toward M1-deactivated, partially M2-polarized macrophages [20]. In a mice model, cigarette smoke extract can induce M2 polarization via protease serine member S31 [21]. Further investigation is required to delineate the effect of cigarette smoking on human immune systems, though our findings of diminished M2a polarization associated with decreased FPR3 expression lighten new insight into targeting FPR2/3 agonist to help resolve neutrophilic inflammation in COPD.

FPR2 is an unconventional receptor for the diversity of its agonists and its ability to convey contrasting biological signals, depending on the ligands engaged on it. It has been identified in various cells types including neutrophils, monocytes, activated T cells, NK cells. LXA4, ANXA1, and RvD1 activates FPR2/FPR2 homodimerization, triggering the p38/MAPKAPK/Hsp27 pathway associated with anti-inflammatory responses, whereas SAA and LL-37 activates FPR2/FPR1 heterodimerization, triggering JNK pathway associated the delay of neutrophil apoptosis and the induction of pro-inflammatory responses [22]. For the first time, we found a link between diminished FPR2 expression on blood neutrophil/Th/Tc cells and the development of COPD or its frequent exacerbation phenotype. In line with our findings, decreased FPR2 expression and LXA4 generation have been recently shown to be involved in a reduction in the ability of inhaled corticosteroids to impair control of airway inflammation in pediatric severe asthma patients [12].

Additionally, FPR 2/LXA4 Receptor can regulate neutrophil-platelet aggregation and attenuate cerebral inflammation after cerebral ischemia/reperfusion injury in a recent murine model [23]. In accordance with our findings that FPR2 expression on helper T cell was increased to a greater extent in COPD patients given oral steroid for more than 3 months, previous studies reported that glucocorticoids are able to induce the expression of FPR2/ALX R and to drive the resolution of inflammation [9].

FPR1 has been shown to be involved on the lung migration of neutrophil and macrophage and pro-inflammatory responses after cigarette smoke exposure in a mouse model [7]. In line with our findings that FPR1 expression on neutrophil was increased in COPD patients with a high MMRC dyspnea scale and reversed to normal after 1-year treatment, previous studies reported that cigarette smoking increased FPR1 numbers on blood neutrophils in emphysema patients, and that its expression was not correlated with the severity of airflow limitation [24, 25].

FPR3 has been implicated in the sensing of infection-associated olfactory cues and in the response to bacterial endotoxin stimuli [26]. Its expression pattern and function are incompletely understood, though FPR3 is thought to function as a decoy receptor which binds to its regular receptor and inhibit its signaling [27]. On the other hand, an acetylated peptide F2L has been found to specifically bind to FPR3, leading to stimulation of intracellular calcium release, inhibition of cAMP production, and activation of the MAP kinases ERK1/2 [28]. Little is known about the relationship between FPR3 expression and COPD. However, it has been proposed that the activation of FPR2/ALX and FPR3 expressed on mature dendritic cells and T cells could, in the later phase of the inflammatory response, sustain the switch from the innate to the physiological adaptive immune response [29]. For the first time, our findings demonstrate that inadequate FPR3 expressions in the immune cells, especially NK cell, may play a role the development of COPD and are correlated with more severe airflow limitation. Notably, inadequate FPR3 expressions on NK T and Th cells in COPD patients could be restored with smoking cessation and oral steroid use, respectively.

It has been demonstrated that pro-resolving and tissue-protective actions of ANXA1-based cleavage-resistant peptides are mediated by FPR2/LXA4 receptor [30]. Targeting the ANXA1-FPR 2 pathway affords protection against bacterial lipopolysaccharide-induced pathologic changes in the murine adrenal cortex [6]. ANXA1-FPR2 counteracts chemokine-induced arterial myeloid cell recruitment, reduces leukocyte-endothelial interactions, and regulates mast cell degranulation [14, 31, 32]. Glucocorticoid-induced leucine zipper gene is involved in dexamethasone-induced inhibition of mouse neutrophil migration via control of ANXA1 expression [33]. For the first time, we found a link between defective ANXA1 protein expression and increased risk of moderate exacerbation in COPD patients. The failure to return to normal range after 1-year follow-up may indicate defective generation or persisted consumption of this endogenous pro-resolving lipid mediator in these patients. In line with our findings, the levels of ANXA1 were found to be low in wheezy infants [11]. Systemic administration of ANXA1 was reported to promote wound recovery in a NADPH oxidase 1-dependent fashion in a mice model [34]. Moreover, ANXA1 is involved in the resolution of neutrophil-mediated inflammatory responses during *Leishmania braziliensis* infection and in murine gout [35, 36]. We hope that our results will lead to novel therapeutic options of using synthetic ANXA1 peptides for COPD in cases where an optimal treatment modality is lacking.

The limitations of our study should be acknowledged. First, the COPD patients and healthy non-smokers were not matched with respect to age, which may potentially contribute to differential FPR expressions. However, identical results were obtained after adjusting these data for age by linear regression model. Second, the cause and effect relationship between FPR1/2/3 expressions and COPD is not straight forward, but the reversal of several altered FPR expressions after 1-year treatment indicate that smoking related FPR1 over-expression and FPR2/3 under-expression could contribute to the airflow limitation in COPD, and the reversal of these imbalance could lead to the improvement in small airway dysfunction. Third, the sample size in the subgroup analysis of the follow-up data is relatively small. However, this provide direct evidence that glucocorticoid can exert it anti-inflammatory effect partly through FPR2/3 up-regulations. Finally, further in vitro study is needed to clarify whether FPR2/3 agonists can serve as novel therapeutic agent to resolve inflammation in COPD.

## Conclusions

Although we acknowledge that the biological and clinical relevance of these findings needs further support by larger studies, our findings indicate that COPD patients are characterized by decreased FPR2/FPR3 expressions and defective ANXA1 generation associated with decreased M2a percentage in the blood immune cells, and a higher moderate exacerbation risk is associated with both decreased FPR2 expression on neutrophil and decreased serum ANXA1 levels. The reversal of these altered FPR1/2/3 expressions and M2a polarization may be involved in the partial improvement in small airway dysfunction after 1-year medical therapy and cessation of smoking.

## Abbreviations

COPD: chronic obstructive pulmonary disease
FPR: formyl peptide receptor
NK: natural killer
Th: T helper
Tc: T cytotoxic
SAA: serum amyloid A
LL37: cathelicidin
LXA4: lipoxin A4
ANXA1: annexin A1
RvD1: resolvin D1
MMRC: modified medical research council
GOLD: global initiative for chronic obstructive lung disease
LABA: long acting β2 agonist
LAMA: long acting muscarinic antagonist
ICS: inhaled corticosteroid
